# Environmental and socioeconomic influences on childhood asthma and pneumonia: burden and healthcare utilization in a population-based cohort study

**DOI:** 10.3389/fmed.2026.1872723

**Published:** 2026-07-15

**Authors:** Fangfang Wang

**Affiliations:** Department of Pediatric, Wuhu Hospital, East China Normal University, Anhui, China

**Keywords:** air pollution, childhood asthma, environmental exposure, pediatric pneumonia, seasonal variability, socioeconomic status

## Abstract

**Introduction:**

Childhood asthma and pneumonia remain major causes of pediatric morbidity worldwide. Their occurrence and healthcare burden are influenced by environmental exposures, socioeconomic conditions, and seasonal variability.

**Objective:**

This study evaluated the disease burden, associated risk factors, and healthcare utilization patterns among children with asthma and/or pneumonia.

**Methods:**

A retrospective cohort study was conducted using pediatric electronic medical records from Wuhu Hospital, Anhui Province, China, covering January 2022 to December 2025. The study included 936 children aged 1–14 years with confirmed diagnoses of asthma, pneumonia, or both conditions. Environmental exposure data, including nitrogen dioxide (NO_2_), particulate matter (PM_2.5_), temperature, and humidity, were obtained from regional monitoring systems. Socioeconomic status was determined using census-based indicators. Multivariable logistic regression was performed to identify factors associated with hospitalization.

**Results:**

Among the 936 children, 47.4% had asthma, 36.5% had pneumonia, and 16.0% had both conditions. Lower socioeconomic status was strongly associated with pneumonia, whereas asthma occurred across all socioeconomic groups. Higher ambient NO_2_ and PM_2.5_ concentrations were significantly associated with asthma, while biomass fuel use and poor household ventilation were linked to pneumonia. Pneumonia incidence peaked during winter, whereas asthma cases increased in spring. Children with pneumonia experienced higher hospitalization rates and longer hospital stays, while asthma was associated with more frequent emergency department visits and outpatient consultations. Recurrent asthma episodes occurred in 44.1% of affected children.

**Discussion:**

Childhood asthma and pneumonia impose a substantial healthcare burden, with distinct environmental, socioeconomic, and seasonal determinants. Strategies aimed at reducing air pollution exposure, improving household air quality through cleaner fuels and better ventilation, and addressing socioeconomic barriers to healthcare access may help reduce disease burden and improve pediatric respiratory health.

## 1 Introduction

Childhood respiratory diseases have been a notable public health concern worldwide. They are a contributor to morbidity, mortality rates and the use of healthcare services among the pediatric populations. Some of these conditions include asthma and pneumonia, which are among the most prevalent and significant respiratory disorders in children. Asthma is a chronic inflammatory airway disease characterized by recurrent wheezing, breathlessness, and airflow obstruction. In contrast, pneumonia is an acute lower respiratory tract infection, which is still the cause of hospitalization and death among children, especially in low- and middle-income countries. The burden of asthma-related hospitalizations and emergency visits requires an in-depth understanding of factors that can affect disease occurrence and healthcare utilization (1). On the same note, pneumonia has remained one of the leading causes of childhood illness across the world, with high prevalence cases being reported in resource-constrained environments and among vulnerable groups (2).

Environmental determinants are also vital to the causes and worsening of respiratory conditions among children. Pediatric asthma has long been known to be triggered and exacerbated by exposure to air pollutants, including particulate matter, nitrogen dioxide, and other atmospheric contaminants (3). Airway swelling, weakened immunity, and reduced resistance to respiratory infections can result from environmental pollution. It has also been found that temporary variations in air quality correlate with a higher number of respiratory illnesses among children, and that there is a direct correlation between environmental exposure and use of healthcare facilities (4). Air pollutants may worsen airway inflammation, dysfunction, and susceptibility to respiratory infections, thereby increasing the risk of severe disease requiring hospitalization. In addition, Barnett-Itzhaki et al. (5) used machine learning models to predict pediatric hospitalizations and identified changes in air pollution levels and humidity as important factors in predicting hospitalization risk. Their results also underscore the significance of environmental exposure in the development of respiratory health outcomes and health care usage among children. Moreover, childhood asthma is a disease much more likely to develop in populations with greater exposure of children to environmental vulnerability at a long-term level (6).

In addition to environmental exposures, the social determinants of health play quite crucial roles in the distribution and outcome of childhood respiratory diseases. Children are exposed to environmental risks and lack of access to healthcare resources because of socioeconomic status, housing conditions, parental education and neighborhood conditions. Children in poor socioeconomic conditions are usually more exposed to pollution and have limited access to preventive health care. All these factors lead to asthma prevalence and worse disease management outcomes. Differences in social determinants are strongly correlated with differences in asthma prevalence and the range of healthcare use among pediatric groups (7). Socioeconomic inequalities are thus a notable dimension in explaining the broader burden of respiratory diseases in children. Environmental and socioeconomic risk factors are also effective interventions in causing childhood pneumonia. The risk factors for pneumonia in young children include exposure to indoor air pollution, insufficient household ventilation, inadequate immunization, and poor nutrition. Hospital-based studies have shown that these determinants are strongly associated with the likelihood and severity of pneumonia among pediatric patients (8). Similarly, epidemiological studies conducted in developing countries have proven that socioeconomic disadvantage and environmental risks are significant contributors to increasing pneumonia prevalence among children below the age of five (9).

Although there is extensive literature on pediatric respiratory diseases, gaps remain in the overall understanding of childhood asthma and pneumonia within an integrated analytical framework. Numerous past studies have examined individual factors associated with these conditions, e.g., environmental exposures, socioeconomic determinants, or healthcare utilization, but not in combination. Subsequently, there is scant evidence on the interplay among environmental factors, socioeconomic differences, and changes over time in determining the burden and patterns of healthcare use for childhood respiratory diseases in the population. Moreover, most of the available literature focuses on either asthma or pneumonia individually, yet the two conditions often share environmental risk factors, healthcare needs, and susceptible population groups. Moreover, few population-based cohort studies have examined the effects of seasonal variability, environmental exposures, and social inequalities on the development of disease patterns and healthcare use among children. To begin with, it is necessary to address these and develop more effective strategies for public health intervention and healthcare planning. The aim of the current research was thus to evaluate the burden, environmental factors, and patterns of health service use for childhood asthma and pneumonia at the population level and to examine how socioeconomic disparities and seasonal variations affect disease prevalence and health service use among children.

## 2 Materials and methods

### 2.1 Study design and setting

To investigate the burden of childhood asthma and pneumonia, factors in the environment that determined these diseases, care patterns, socioeconomic differences, and seasonal changes, a retrospective, population-based cohort study was carried out. The study was conducted in Wuhu, Anhui Province, China. Pediatric healthcare data were obtained retrospectively from Wuhu Hospital and its affiliated pediatric outpatient and emergency care services between January 2022 and December 2025. The design enabled longitudinal evaluation of patterns in respiratory disease, environmental exposures, and healthcare use among children in the selected population.

The study period from January 2022 to December 2025 was chosen because it had full EMR, environmental monitoring, and socioeconomic data at the time of data extraction for the most recent 4-year period. To discriminate seasonal variation, healthcare utilization patterns, and recurrent respiratory outcomes while minimizing the effects of short-term fluctuations, a 4-year observation period was deemed adequate for assessing temporal trends.

Contemporary epidemiological methods in environmental health research, involving the investigation of the burden and determinants of respiratory disease amongst pediatric populations, informed the study design (1, 10, 11). The integration of clinical information with selected environmental exposure indicators and socioeconomic variables enabled evaluation of key factors associated with respiratory disease burden, temporal disease patterns, and healthcare utilization among study participants.

### 2.2 Study population and sample size

The research population included children aged 1–14 years diagnosed with asthma, pneumonia, or both between January 2022 and December 2025. 936 pediatric patients were selected by reviewing hospital electronic medical records, outpatient clinics registries, and pediatric emergency department databases.

The age group of 1–14 years was chosen because it encompasses the most significant pediatric developmental phases during which asthma and pneumonia account for a large proportion of respiratory system morbidity. Children under the age of 1 year were excluded as respiratory illnesses in infancy have different clinical entities, diagnostic criteria, and risk factors than do those in older children. The age range was capped at 14 years, as children of this age are regularly seen in the study institution’s pediatric services, thereby ensuring consistency in diagnostic and healthcare records. Epidemiological studies of respiratory diseases among children have been conducted, using similar age groupings (12, 13).

A population-based cohort was used to include inpatient and outpatient cases, capturing all degrees of disease severity and levels of healthcare utilization. The analysis included all available cases that met all conditions and had full clinical and demographic information at the time of the study. The cohort method facilitated the determination of changes in disease prevalence over time and enabled comparison of results between the socioeconomic and environmental exposure groups.

### 2.3 Inclusion criteria

Children were included in the study if they met specific criteria. Eligible participants were those aged 1–14 years during the study period who had an established clinical diagnosis of asthma or pneumonia, documented in either inpatient or outpatient records in accordance with recognized pediatric diagnostic standards. In addition, the children were required to reside within the defined geographic study area. Only cases with complete medical records were considered, including demographic information, diagnostic details, and health care utilization data.

### 2.4 Exclusion criteria

Children were not included if their medical histories were incomplete, if there was no information on diagnosis, or if the information was unclear. Patients with chronic respiratory diseases not due to asthma or pneumonia, including congenital lung malformations, cystic fibrosis, or severe immunodeficiency diseases, were also excluded, as these conditions can independently affect respiratory morbidity and healthcare use patterns. Also, the children who had not been living in the study area were not observed, making them ineligible for assessment of environmental exposure.

### 2.5 Data sources and data collection

Various sources were accessed retrospectively to obtain data on all aspects of clinical outcomes, environmental exposures, and socioeconomic characteristics. The clinical data were extracted from the electronic medical record system of Wuhu Hospital, including the emergency department, outpatient clinic, and pediatric inpatient databases. Other demographic and public health data were provided by the regional public health surveillance systems in Wuhu, Anhui Province, China. Demographic data, diagnoses, treatment, hospitalizations, outpatient consultations, emergency department visits, and length of stay were gathered from these sources.

Information on environmental exposures was derived at both the personal and regional levels. Household exposures that impact health, such as tobacco smoke exposure, indoor biomass fuel use, ventilation, and pets, were collected from the household, at clinic intake, and from community health records in the integrated health information system. Furthermore, data on ambient air pollutants (NO_2_, particulate matter) and climatic parameters (humidity, temperature, seasonal rainfall) were obtained from the regional environmental monitoring network and meteorological databases. Ambient air pollution data were regularly collected from the region’s environmental monitoring network and meteorological monitoring systems in Anhui Province. The methods used to assess environmental exposures were based on previous environmental epidemiology studies of pediatric respiratory outcomes (10, 11). These exposure estimates were associated with patients’ residence and were therefore used to estimate environmental exposures before they were diagnosed or hospitalized.

Administrative census databases and area-based socioeconomic indices linked to residential addresses were used to obtain socioeconomic information. Household factors such as the household income category, parental education level, housing, crowding, and access to healthcare services were considered variables. Information regarding active smoking by study participants was not routinely recorded in the pediatric electronic medical records and was therefore unavailable for analysis.

No questionnaires were given specifically for this study. All household environmental information was obtained by screening existing intake assessment forms and community health records collected during routine clinical care, which was a retrospective approach.

Pre-analysis validation of data completeness was performed. Missing data rates for all household environmental variables were below 10%. Because missingness was low, complete-case analysis was considered appropriate for multivariable regression models. Participants with missing values for variables included in a specific model were excluded from that model.

#### 2.5.1 Environmental and household exposure data quality

The frequency of clinical encounter records containing environmental and household exposure variables was inconsistent across all patients. Accordingly, data on smoking in the home, household biomass fuel use, household air exchange, and presence of pets inside the home were obtained from the linked pediatric intake assessments and community health records, where available. Before analysis, data quality checks were performed to ensure consistency of records. Multivariable analyses of these exposure variables were limited to cases with complete data for the variables being analyzed. Given the low proportion of missing data ( < 10% across all household exposure variables), a complete-case analysis was unlikely to affect model estimates substantially. Results were robust to sensitivity analyses excluding incomplete records.

### 2.6 Key variables operational definitions

#### 2.6.1 Childhood asthma

Childhood asthma was identified as physician-confirmed in hospital or outpatient records of asthma diagnoses, according to accepted pediatric diagnostic criteria, such as frequent wheezing, airway obstruction, and response to bronchodilator medication.

#### 2.6.2 Pneumonia

Pneumonia was defined as a clinically diagnosed lower respiratory tract infection with fever, cough, and pain, and radiological or clinical evidence of lung inflammation documented in patients’ medical records.

#### 2.6.3 Environmental exposure

Environmental exposure was defined as the concentration of pollutants in the ambient air and the climate at the place of residence of individual patients. The key environmental indicators were nitrogen dioxide concentration, particulate matter levels, ambient temperature, humidity, and seasonal climatic fluctuations.

#### 2.6.4 Health service utilization

The use of health services was determined as the frequency and nature of health services used by children to manage asthma or pneumonia. Hospital admissions, emergency department visits, outpatient consultations and length of hospital stay were considered indicators.

#### 2.6.5 Seasonal variability

Seasonal variability refers to variation in disease rates and health care utilization across seasons in a year. Each case was classified by the month and meteorological season in which the diagnosis or hospitalization was made.

### 2.7 Evaluation of environmental determinants

Environmental determinants were assessed using routinely collected data from regional environmental monitoring stations and meteorological surveillance systems. Exposure assessment methods were based on approaches commonly used in environmental epidemiology studies investigating pediatric respiratory health outcomes (10, 11). Key indicators included ambient air pollutants, particularly nitrogen dioxide (NO_2_) and particulate matter (PM_2.5_), as well as climatic variables such as temperature and humidity (10). Exposure estimates were obtained using environmental monitoring stations within the study area, located near patients with residential proximity. Ambient NO_2_ and PM_2.5_ concentrations for each year were assigned based on residential proximity, using monitoring network data. The exposure categories were defined based on the study population’s exposure distribution by dividing it into three tertiles. The participants were assigned to low, medium, and high exposure groups based on the lowest, median, and highest third of exposure levels for both NO_2_ and PM_2.5_.

In addition to air pollution, climatic factors such as temperature, humidity, and seasonal conditions were also included in the analysis. These environmental indicators were added to assess the probability of environmental stimuli for asthma exacerbation and the incidence of pneumonia. The level of environmental exposure was assigned to each participant based on the distance from their residential address to the nearest environmental monitoring station, so that ambient pollutant levels can be approximated before they are diagnosed or hospitalized.

### 2.8 Socioeconomic status assessment

Socioeconomic status was measured using an area-based index derived from census-based indicators for patients’ residential areas. Some of the variables included in the index were household income group, the level of parental education, residential density, and housing conditions. Composite scores were used to classify patients into low-, middle-, and high-socioeconomic groups. Such categorization facilitated the assessment of differences in the burden of disease, health care use, and patterns of environmental exposures across socioeconomic classes, as is in line with public health models of the social determinants of respiratory disease in pediatrics (1).

The distance from the participant’s home to the nearest healthcare facility was grouped as < 5 km or ≥ 5 km. We chose the 5 km threshold because it was considered a practical measure of geographic access from the distribution of residential distances among residents in the study and from other studies of the geographic accessibility of healthcare facilities, that a distance of 5 km or more could be a barrier to health care use, especially for vulnerable groups (14, 15).

### 2.9 Seasonal variability analysis

The seasonal change in the number of cases was determined by dividing cases by month and by the meteorological season of diagnosis or hospitalization. Four seasons were used to divide the calendar year to enable the analysis of the temporal movements. The distribution of asthma exacerbation and pneumonia cases on a monthly and seasonal basis was studied to determine whether there were seasonal peaks in disease occurrence. There have been earlier epidemiological reports of seasonal changes in respiratory disorders among the pediatric population related to environmental and climatic variations (16, 17).

### 2.10 Outcome measures

The primary study outcome was the overall burden of childhood asthma and pneumonia in the cohort population. The burden of disease was assessed using the number of diagnosed cases and the frequency of hospitalizations observed within the cohort during the study period. The secondary outcomes were the associations between environmental determinants and the occurrence of respiratory diseases, seasonal variation in disease presentation, and disparities in healthcare use across socioeconomic groups. Other results included the recurrence of asthma attacks, pneumonia-related hospitalizations, and healthcare utilization patterns over the course of the study.

### 2.11 Statistical analysis

All statistical analyses were performed using standard epidemiological software. The general characteristics of the cohort (demographic characteristics, environmental exposures, and healthcare utilization patterns) were summarized using descriptive statistics. Means and standard deviations were used to describe continuous variables, and frequencies and percentages were used to describe categorical variables.

Univariate logistic regression analyses were first conducted to estimate crude odds ratios (ORs) and 95% confidence intervals (CIs) for each environmental and socioeconomic factor and for hospitalization for respiratory disease. Univariate analysis was performed, followed by multivariable analysis of variables identified by univariate analysis, adjusted for age and sex. Crude and adjusted ORs and 95% CIs were presented. Adjusted odds ratios (ORs) and confidence intervals (CIs) were estimated to assess the strength of the associations between determinants and the risk of hospitalization. The time-series analysis was applied to assess monthly variations in disease incidence and seasonal differences in the study period. Sensitivity analyses comparing participants with complete and incomplete environmental exposure data demonstrated similar demographic and clinical characteristics, suggesting minimal impact of missingness on model stability. Regression models were used to examine whether an interaction between environmental exposures and socioeconomic status existed and included interaction terms.

### 2.12 Recurrent asthma and time-to-event analysis

To assess the recurrence of asthma exacerbations over the follow-up period, a time-to-event analysis was conducted among children diagnosed with asthma. Recurrence was prescribed as a follow-up asthma attack necessitating medical attention to the initial diagnosis within the research process. The probability of remaining asthma-free was estimated using Kaplan-Meier survival analysis. The log-rank test was used to compare differences in recurrence patterns across environmental exposure and socioeconomic groups. The calculated follow-up period was from the date of the first asthma diagnosis to the date of recurrence or termination of the study.

### 2.13 Ethical considerations

The study adhered to the principles stated in the Declaration of Helsinki. This research was approved by the Ethics Committee of Wuhu Hospital (approval number: LC20250628), and the need for informed consent was waived due to the retrospective nature of the study. All participant data were identified before analysis, and no images of identifiable individuals are included in the manuscript.

## 3 Results

### 3.1 Baseline demographic and clinical characteristics

During the study period from January 2022 to December 2025, a total of 936 pediatric patients diagnosed with asthma and/or pneumonia were included in the final cohort analysis. The age distribution indicated that the majority of cases occurred among school-age children (5–9 years), followed by preschool and adolescent groups. Male participants constituted a slightly higher proportion of the cohort, consistent with reported epidemiological patterns of pediatric respiratory diseases.

Asthma represented the most common diagnosis in the cohort, while a considerable proportion of acute respiratory presentations were attributed to pneumonia. A smaller subset of children experienced both asthma and pneumonia episodes during the study period. Environmental exposures such as household smoking and overcrowding were frequently reported, highlighting the potential role of environmental risk factors in respiratory disease burden among children. Table 1 presents the baseline demographic, clinical, and household characteristics of the entire study cohort (*n* = 936). Because these variables are presented descriptively for the full cohort rather than as comparisons between groups, formal statistical hypothesis testing was not performed. In addition, previous respiratory hospitalizations and recurrent wheezing episodes were observed in several participants, indicating that respiratory morbidity in this population may often be chronic or recurrent.

**TABLE 1:** Baseline demographic, clinical, and household characteristics of the study cohort (*n* = 936).

Variable	Category	Frequency (*n*)	Percentage (%)
Age group	1–4 years	294	31.4
	5–9 years	368	39.3
	10–14 years	274	29.3
Sex	Male	506	54.1
	Female	430	45.9
Primary diagnosis	Asthma	444	47.4
	Pneumonia	342	36.5
	Both conditions	150	16.0
Residential area	Urban	472	50.4
	Peri-urban	286	30.6
	Rural	178	19.0
Parental education level	Primary or less	298	31.8
	Secondary	402	42.9
	Higher education	236	25.2
Household crowding	≤ 3 persons/room	508	54.3
	> 3 persons/room	428	45.7
Household smoking exposure	Yes	322	34.4
	No	614	65.6
History of recurrent wheezing	Yes	362	38.7
	No	574	61.3
Previous hospitalization for respiratory illness	Yes	206	22.0
	No	730	78.0

### 3.2 Socioeconomic status and distribution of respiratory diseases

the analysis of socioeconomic status revealed notable differences in the distribution of respiratory diseases among children. Pneumonia was more frequently observed in children from lower socioeconomic households, where environmental risk factors such as household crowding and exposure to indoor smoke were also more prevalent. In contrast, asthma cases were reported across all socioeconomic groups, with a relatively balanced distribution among middle-income families.

As presented in Table 2, children from economically disadvantaged households experienced a greater burden of pneumonia, suggesting that socioeconomic deprivation may increase vulnerability to infectious respiratory diseases. Parental education level was also associated with disease patterns, with lower educational attainment corresponding to higher pneumonia prevalence. Additionally, healthcare utilization patterns appeared to be influenced by socioeconomic conditions, particularly access to healthcare facilities and insurance coverage, indicating that socioeconomic factors may affect both disease occurrence and access to medical care. Both frequencies and row percentages are shown to facilitate comparison of respiratory disease distribution across socioeconomic groups (Table 2).

**TABLE 2:** Distribution of respiratory diseases across socioeconomic indicators.

Scioeconomic indicator	Category	Asthma n (%)	Pneumonia n (%)	Both conditions n (%)	Total
Household income level	Low	128 (35.4)	170 (47.0)	64 (17.7)	362
	Middle	198 (54.7)	112 (30.9)	52 (14.4)	362
	High	118 (55.7)	60 (28.3)	34 (16.0)	212
Parental education	Primary	120 (40.3)	128 (43.0)	50 (16.8)	298
	Secondary	192 (47.8)	152 (37.8)	58 (14.4)	402
	Higher	132 (55.9)	62 (26.3)	42 (17.8)	236
Health insurance coverage	Yes	262 (49.8)	188 (35.7)	76 (14.4)	526
	No	182 (44.4)	154 (37.6)	74 (18.0)	410
Access to healthcare facility	< 5 km	286 (49.0)	210 (36.0)	88 (15.1)	584
	≥ 5 km	158 (44.9)	132 (37.5)	62 (17.6)	352

### 3.3 Environmental exposure characteristics

Environmental exposure analysis involved both the indoor and outdoor environmental determinants. The children who lived in regions with high air pollution or near high-traffic roads had a higher rate of asthma diagnoses and respiratory symptoms. Table 3 indicates the environmental conditions in relation to the outcome of respiratory diseases. An increased incidence of asthma was observed among children exposed to moderate-to-high levels of ambient air pollutants, suggesting a possible environmental trigger for airway inflammation. The presence of indoor environmental influences on pneumonia cases, including biomass fuel consumption and poor ventilation, was found to be stronger, reflecting the effects of indoor air quality on respiratory diseases among children.

**TABLE 3:** Environmental exposure indicators among children with respiratory diseases.

Environmental factor	Category	Asthma (n)	Pneumonia (n)	Both (n)
Ambient NO_2_ exposure	Low	132	108	36
	Moderate	176	138	58
	High	136	96	56
PM_2.5_ exposure level	Low	120	98	32
	Moderate	184	148	58
	High	140	96	60
Distance to major road	< 500 m	226	170	72
	≥ 500 m	218	172	78
Indoor biomass fuel use	Yes	118	152	60
	No	326	190	90
Household ventilation	Adequate	268	166	72
	Limited	176	176	78
Presence of indoor pets	Yes	142	84	40
	No	302	258	110

Ambient NO_2_ and PM_2.5_ exposure categories were defined using cohort-specific tertiles of annual average pollutant concentrations, assigned based on residential proximity to monitoring stations.

### 3.4 Seasonal distribution of respiratory diseases

Seasonal analysis demonstrated clear temporal variation in the incidence of respiratory diseases among children. Pneumonia cases were most prevalent in the colder winter months, while asthma exacerbations tended to increase during transitional seasons with fluctuating temperatures and humidity. Monthly trend analysis further indicated that respiratory admissions were slightly elevated during late winter and early spring.

As shown in Table 4, pneumonia cases peaked during winter, consistent with typical seasonal patterns of respiratory infections. In contrast, asthma exacerbations occurred throughout the year but showed a modest increase during spring, which may be associated with environmental triggers such as pollen exposure and climatic changes. Table 4 presents both frequencies and row percentages, illustrating the relative distribution of respiratory diseases across seasons.

**TABLE 4:** Monthly and seasonal distribution of asthma and pneumonia cases.

Season	Asthma n(%)	Pneumonia n(%)	Both n(%)	Total
Winter	120 (38.5)	150 (48.1)	42 (13.5)	312
Spring	138 (53.9)	82 (32.0)	36 (14.1)	256
Summer	88 (50.0)	58 (33.0)	30 (17.0)	176
Autumn	98 (51.0)	52 (27.1)	42 (21.9)	192

Percentages are calculated within each season (row percentages).

To have an additional illustration of the temporal distribution of respiratory illnesses during the study period, monthly variations in asthma and pneumonia were studied. The graphical analysis illustrates that there are specific seasonal variations with the cases of pneumonia being more prevalent during colder seasons and asthma exacerbations having peaks during the transitional seasons when there is a change in temperature and humidity.

**FIGURE 1:**
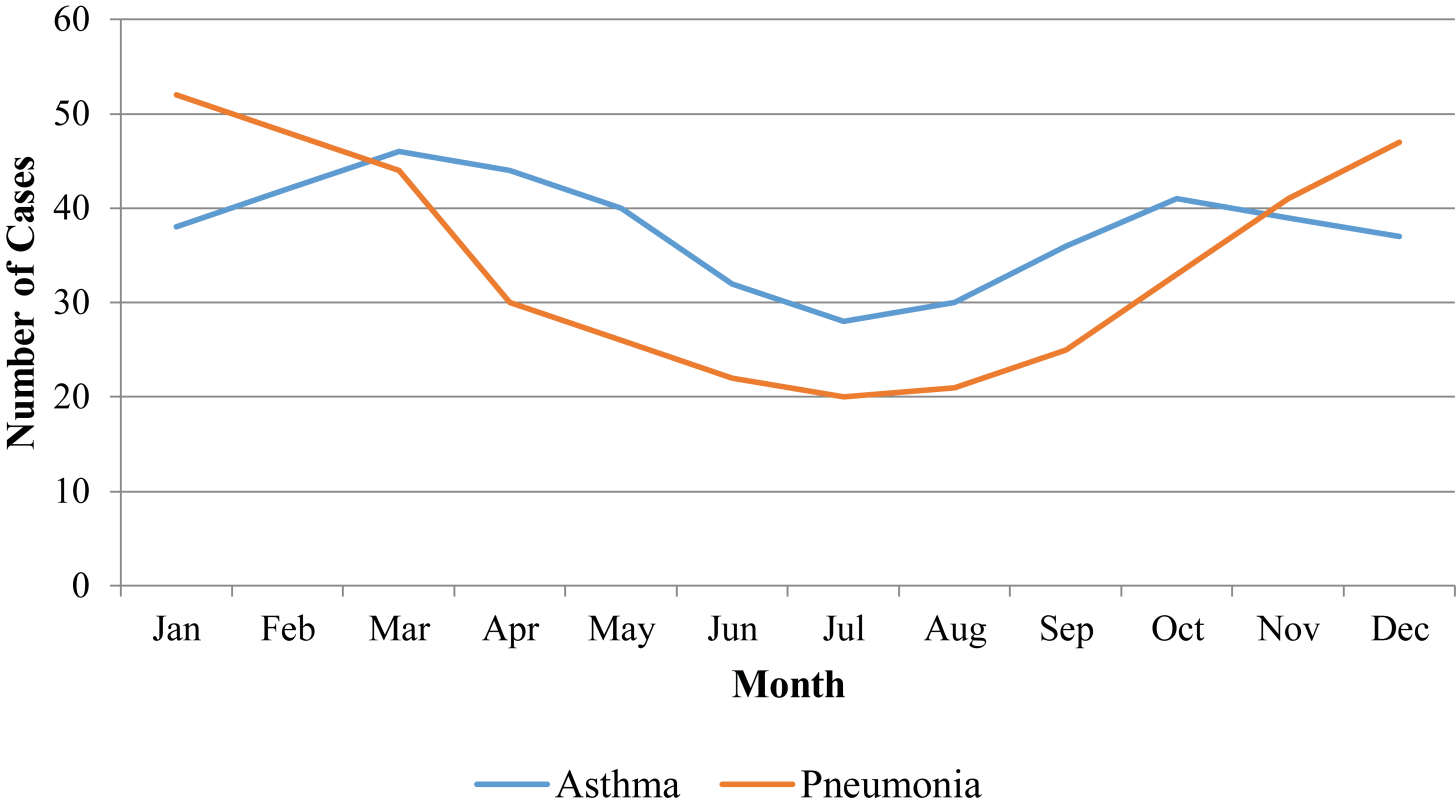
Seasonal trend of pediatric respiratory cases.

The line chart in Figure 1 represents the change in number of asthma and pneumonia cases diagnosed every month among the cohort of children. The cases of pneumonia exhibit definite peaks in the winter months, but the cases of asthma exacerbation exhibit moderate increments in the transitional seasons of spring and autumn. The trend indicates that climatic conditions and environmental determinants could be used as contributing factors to seasonal changes in the burden of pediatric respiratory diseases.

### 3.5 Health service utilization patterns

The analysis of healthcare utilization revealed distinct patterns between asthma and pneumonia cases. Pneumonia was more frequently associated with hospital admissions and longer inpatient stays, reflecting the severity of acute lower respiratory infections. In contrast, asthma cases were more commonly managed through emergency department visits and outpatient services.

As shown in Table 5, the higher hospitalization rate among pneumonia patients indicates the need for inpatient management and monitoring. Conversely, asthma-related healthcare utilization was concentrated in emergency and outpatient settings, consistent with the chronic and episodic nature of asthma management.

**TABLE 5:** Healthcare utilization patterns among children with respiratory diseases.

Utilization indicator	Asthma	Pneumonia	Both
Hospital admissions	172	214	82
Emergency department visits	246	188	90
Outpatient visits	362	218	104
Repeat hospitalizations	64	82	40
Average hospital stay (days)	3.6	5.0	5.4
ICU admissions	18	28	12

### 3.6 Multivariable analysis of environmental and socioeconomic determinants

Univariate logistic regression models were first employed to generate crude estimates of association between environmental and socioeconomic factors and hospitalization for respiratory disease. Variables that were associated significantly were then added to the multivariable model. Ambient air pollution exposure (both crude and adjusted) and household smoking exposure, biomass fuel use, low socioeconomic status, winter season diagnosis, and previous respiratory hospitalization were identified as significant predictors of hospitalization in both crude and adjusted analyses, as reflected in Table 6. The associations were mostly persistent following age and sex adjustment, suggesting that environmental and socioeconomic factors contribute to respiratory disease burden independently of each other.

**TABLE 6:** Crude and adjusted logistic regression analysis of determinants associated with respiratory disease hospitalization.

Variable	Category	Crude OR (95% CI)	Adjusted OR (95% CI)	*p*-value
High ambient NO_2_ exposure	vs. low	1.55 (1.18–2.04)	1.42 (1.08–1.86)	0.01
High PM_2.5_ exposure	vs. low	1.48 (1.12–1.95)	1.36 (1.02–1.78)	0.03
Household smoking exposure	Yes vs. No	1.62 (1.23–2.13)	1.48 (1.12–1.96)	0.005
Indoor biomass fuel use	Yes vs. No	1.68 (1.27–2.22)	1.52 (1.14–2.04)	0.004
Limited household ventilation	vs. adequate	1.44 (1.09–1.89)	1.34 (1.01–1.78)	0.04
Low socioeconomic status	Vs. high	1.74 (1.32–2.30)	1.58 (1.18–2.12)	0.002
Distance to healthcare facility ≥ 5 km	vs. < 5 km	1.33 (1.00–1.77)	1.26 (0.94–1.69)	0.08
Winter season diagnosis	vs. summer	1.58 (1.20–2.09)	1.46 (1.09–1.95)	0.01
Previous respiratory hospitalization	Yes vs. No	1.91 (1.39–2.62)	1.72 (1.24–2.38)	0.001

OR, odds ratio; CI, confidence interval; NO_2_, nitrogen dioxide; PM_2.5_, particulate matter ≤ 2.5 μm.

The results indicated that exposure to ambient air pollutants, indoor smoke exposure, and low socioeconomic status were among the strongest predictors of respiratory disease hospitalization. Seasonal variation also contributed to disease burden, with diagnoses occurring during winter showing higher odds of hospitalization. In addition, children with a history of previous respiratory hospitalization demonstrated an increased likelihood of readmission, suggesting persistent vulnerability to respiratory illnesses.

High ambient pollutant exposure refers to the highest tertile of annual average NO_2_ or PM_2.5_ concentration compared with the lowest tertile. Crude ORs were obtained from univariate logistic regression models. Adjusted ORs were estimated from multivariable logistic regression models controlling for age and sex.

**FIGURE 2:**
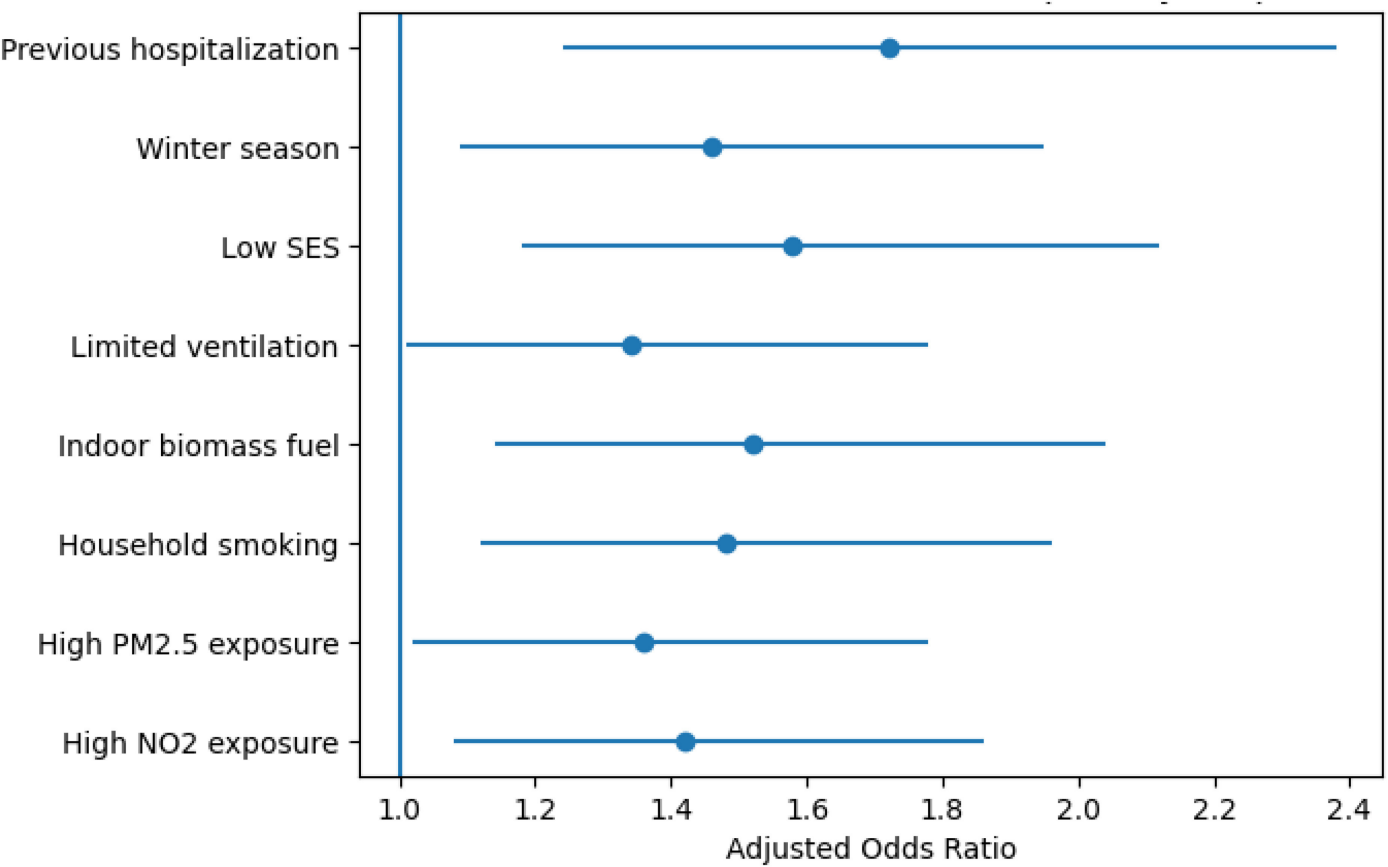
Determinants associated with pediatric respiratory hospitalization.

Relative strength of the environmental and socioeconomic determinants related to respiratory disease hospitalization is demonstrated in the forest plot provided in Figure 2. The data displays a modified odds ratio and confidence interval of environmental exposures and socioeconomic indicators after eliminating the impact of age and sex. Exposure to more air pollution in the ambient environment, smoking tobacco in the home, the use of biomass fuel in the home, and poor ventilation of the home were associated with increased risks of hospitalization in children. There was also a high risk of hospitalization when socioeconomic deprivation and previous respiratory hospitalization were involved.

### 3.7 Recurrent asthma episodes and time-to-event analysis

The longitudinal cohort analysis evaluated the occurrence of recurrent asthma exacerbations during the study period from January 2022 to December 2025. Recurrent episodes were defined as asthma exacerbations requiring medical care following the initial diagnosis within the study timeframe. Among children diagnosed with asthma, a substantial proportion experienced at least one recurrent exacerbation during follow-up. Recurrence rates varied across environmental exposure and socioeconomic groups. Children exposed to higher levels of ambient air pollution demonstrated a greater likelihood of recurrent asthma exacerbations. Similarly, household environmental conditions played an important role, as poor ventilation and exposure to indoor biomass fuel and tobacco smoke were associated with shorter intervals between exacerbations. Socioeconomic disparities were also evident, with children from lower socioeconomic backgrounds experiencing more frequent and earlier recurrences.

Time-to-event analysis was conducted to estimate the probability of remaining free from recurrent asthma exacerbations during follow-up. The Kaplan–Meier analysis was conducted across the available follow-up period of up to 48 months, depending on the date of cohort entry. The survival curves demonstrated a gradual decline in recurrence-free probability over time.

As summarized in Table 7, nearly half of the children with asthma experienced at least one recurrent exacerbation. Most recurrences occurred within the first 6 months after the initial diagnosis, indicating a period of increased disease instability and vulnerability to environmental triggers. A smaller subgroup experienced multiple recurrent episodes, reflecting persistent or inadequately controlled asthma. These recurrent exacerbations frequently resulted in emergency department visits and, in some cases, hospital admissions, highlighting the substantial healthcare burden associated with repeated asthma flare-ups.

**TABLE 7:** Recurrent asthma episodes and follow-up outcomes among children with asthma (*n* = 444).

Variable	Category	Frequency (n)	Percentage (%)
Recurrence of asthma episodes	No recurrence	248	55.9
	≥ 1 recurrent episode	196	44.1
Number of recurrent episodes	One episode	122	27.5
	Two episodes	52	11.7
	≥ 3 episodes	22	5.0
Time to first recurrence	≤ 3 months	68	15.3
	4–6 months	72	16.2
	7–12 months	38	8.6
	> 12 months	18	4.1
Recurrence requiring hospitalization	Yes	64	14.4
	No	132	29.7
Recurrence requiring emergency visit	Yes	104	23.4
	No	92	20.7

**FIGURE 3:**
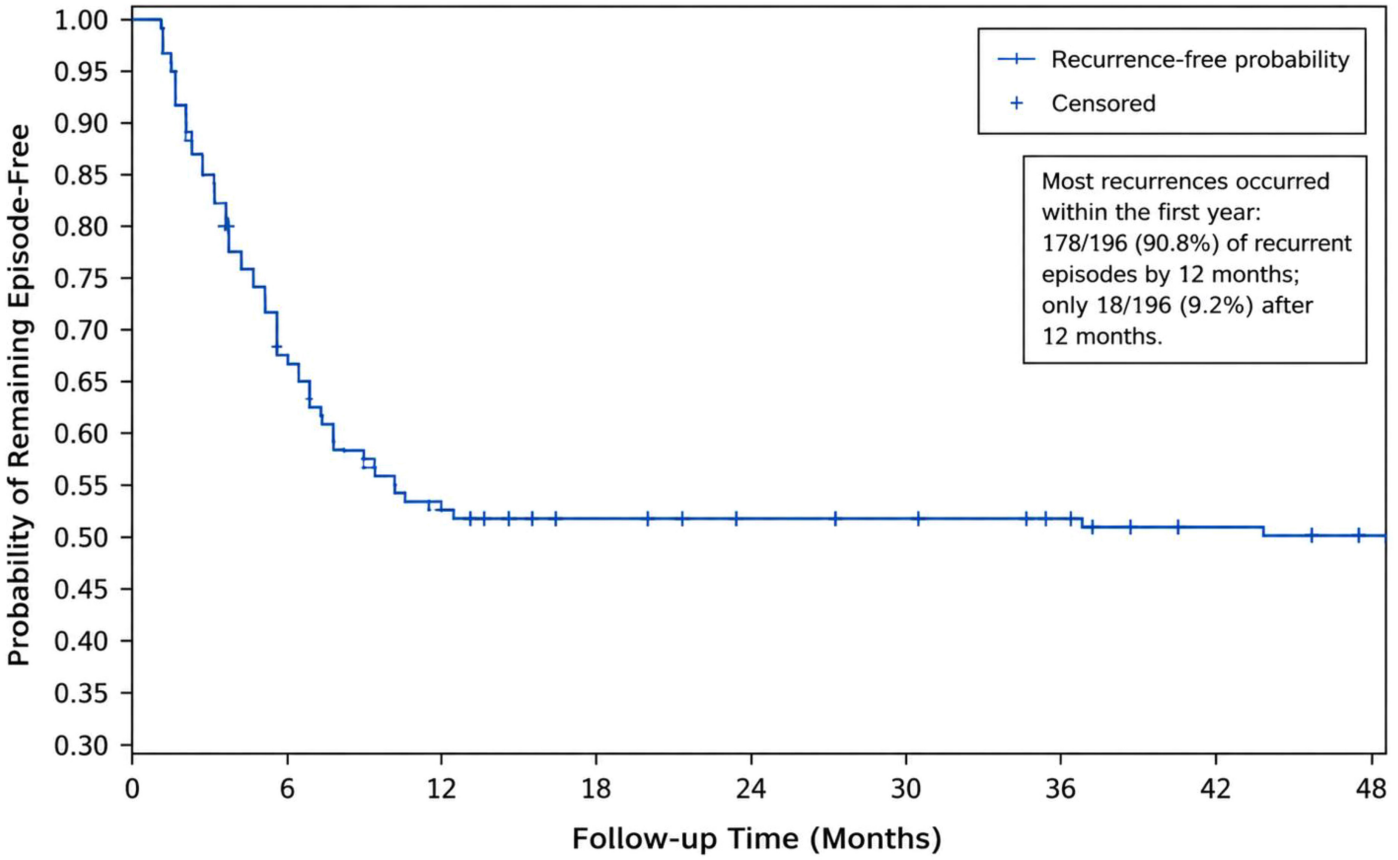
Kaplan-Meier curve for recurrent asthma episodes.

The likelihood of staying free of recurrent asthma exacerbation over the follow-up was assessed with the help of Kaplan-Meier survival analysis. Survival curve is the likelihood of not experiencing recurrent cases of asthma with time. There was a progressive reduction of recurrence free probability and the high risk of recurrence was noted in the early months of the first diagnosis. Children who were exposed to unfavorable environments and socioeconomic conditions recorded shorter intervals of recurrence (Figure 3).

The probability of remaining free from recurrent asthma exacerbation declined most rapidly during the first year after diagnosis, reflecting the concentration of recurrence events within the early follow-up period. Thereafter, the curve plateaued, indicating relatively few additional recurrence events during extended follow-up of up to 48 months.

## 4 Discussion

The burden of respiratory diseases in children with asthma and pneumonia was high, with the largest proportion observed among school-aged children (5–9 years). This age distribution aligns with the epidemiological distribution worldwide, indicating that respiratory morbidity tends to rise during the school years due to increased exposure to environmental triggers and infectious agents, as well as fluctuating immune responses. In all regions of the world, school-aged children (5–14 years) remain a significant group with a burden of respiratory morbidity and asthma, emphasizing the need for respiratory disease prevention in school-aged children (12). Similarly, Melén et al. (13) reported asthma prevalence as being extremely high during school age, and it is frequently observed during early childhood, due to environmental exposure throughout childhood and childhood genetic susceptibility.

There are also studies showing that the prevalence of asthma is greater in boys than girls during childhood, primarily due to the anatomy of the airways, hormones and immune system differences between the sexes (18). Similarly, a pattern of increased prevalence of asthma and recurrent wheezing disorders in young children was observed among males, as reported by Muther et al. (19). The results of this study indicate that susceptibility to childhood respiratory diseases remains associated with biological gender.

Asthma is the most common chronic respiratory condition in children around the world, and pneumonia is the biggest cause of hospitalization and mortality in children, especially in low- and middle-income countries. Chronic respiratory diseases and acute respiratory infections still co-exist and are reported to be important causes of childhood morbidity in developed and developing health care environments alike (20).

The high rates of environmental exposures, repeated episodes of wheezing and previous hospitalizations reported in this study underscore the chronic and multifactorial nature of childhood respiratory diseases. Biological susceptibility, environmental exposures and social determinants of childhood respiratory health all play a role in determining the pattern of disease over time. In the international literature, environmental conditions, socioeconomic disadvantage, and healthcare accessibility have been identified as among the most important determinants of respiratory health outcomes throughout childhood and adolescence. Bush et al. (21) stressed that social determinants have influenced respiratory health since birth and are a key driver of respiratory inequalities across diverse populations worldwide.

In the current study, the authors showed significant socioeconomic differences in the prevalence of respiratory diseases, with pneumonia more prevalent among children from the poorest families and those with lower parental education levels. They are consistent with considerable evidence showing that socioeconomic disadvantage is one of the strongest determinants of poor respiratory health outcomes in children. A study by Gaffney et al. (22) documented that, over the decades, disadvantaged groups in the United States had higher disease burdens and worse respiratory health outcomes. These patterns have also been seen in many other countries, which indicates that poverty, poor housing, and lack of health care resources are universal risk factors for childhood respiratory disease.

Children with pneumonia might have lower parental education levels, which could reflect differences in health literacy, health care seeking, and preventive health care practices. Similar results have been found in other studies in low- and middle-income areas where parent education has a significant impact on the recognition of respiratory symptoms, adherence to vaccination schedules, and the timely use of health services. The study has shown that social determinants of the progression and management of pneumonia in rural Bangladesh were associated with poorer outcomes among children hospitalized with pneumonia, underscoring the critical role of these determinants in the disease process (23).

The present study’s patterns of health care delivery also highlight the significance of structural determinants of respiratory health. Overall, children living near health services and those with health insurance were more likely to use them, suggesting that the availability of health services may be a barrier to disease outcomes. Such differences have been reported in the U.S., with disparities in care access linked to changes in acute care use among children with asthma (24).

The results of environmental exposures analysis revealed significant relationships between respiratory diseases and ambient air pollution, biomass fuel use and inadequate household ventilation, both outdoor and indoor environmental exposures. Both higher exposure to NO_2_ and PM_2.5_ were associated with a higher number of diagnosed asthma cases and respiratory symptom scores among children, highlighting the important contribution of environmental pollution to childhood respiratory morbidity. These results are similar to those reported in several other countries, which have shown a strong association between outdoor air pollution and asthma and wheezing in children. Airway inflammation, oxidative stress and impaired lung development have been consistently linked to traffic-related air pollutants, both in the development and exacerbation of respiratory diseases (25).

Seasonal analysis showed significant temporal variation in respiratory disease occurrence, with the highest number of pneumonia cases in winter and the highest number of asthma cases in transitional seasons. This has been noted in other parts of the world as well. In China, Wu et al. (26) reported higher circulation of respiratory pathogens among children during the colder months and found that this led to higher rates of respiratory infections. Similarly, a recent systematic review and meta-analysis showed that extreme temperatures are significant risk factors for pneumonia in children, suggesting that climate affects the incidence and severity of pneumonia (27).

The current study further suggests that disease severity and health care utilization may be affected by environmental and climatic conditions. The same has been found in Bangladesh, where a record of climate variability was linked to the length of hospital stay for children admitted with pneumonia (23). Worldwide, asthma is a major cause of ED visits among children, especially when environmental exposure and respiratory infections are higher. Research in the U.S. has shown that asthma continues to be a significant contributor to emergency health care use in children (28). In addition, the overlap between asthma and pneumonia diagnoses has been observed more frequently in clinical practice. Leyenaar et al. (29) reported significant differences in diagnoses and prognoses among children with features of both asthma and pneumonia, highlighting the difficulty of differentiating these conditions in hospitalized children. The findings from these observations highlight the need for accurate diagnosis and timely action to improve children’s respiratory outcomes (30).

Mechanisms linking these associations to biology are oxidative stress, airway inflammation, and immune system dysfunction due to exposure to environmental pollutants. Experimental and clinical data suggest that air pollutants activate inflammatory pathways in the respiratory tract, thus making people more susceptible to infections and worsening chronic respiratory diseases. Dondi et al. (31) concluded that oxidative stress is a major pathway linking outdoor air pollution to childhood respiratory morbidity. In addition, epidemiological research in China has found that air pollution has an immediate effect on children’s health, as short-term increases in air pollution have been linked to a dramatic increase in outpatient visits for children’s respiratory diseases (32).

The combination of adverse indoor and outdoor environments and socioeconomic deprivation may increase respiratory health risks through greater exposure to pollutants and reduced access to preventive and curative health care. A recent systematic review and meta-analysis found that children, pregnant women, and other vulnerable groups face an elevated risk of hospitalization due to pneumonia exposure, both short- and long-term (33). In a similar study, the researchers found that the combined effect of environmental pollution and unfavorable socio-environmental conditions causes poor respiratory health outcomes in children, especially among the socioeconomically disadvantaged population in India (34). The results are consistent with the notion that childhood respiratory diseases are not caused by a single risk factor, but rather by a combination of environmental, social and healthcare-related factors.

### 4.1 Strengths of the study

There are a few critical strengths of this study that contribute to the addition of this study to the field of pediatric respiratory epidemiology. First, the retrospective cohort design was based on population that made it possible to analyze a significant number of pediatric cases in the course of 4 years and determine the trends in diseases and the patterns of their recurrence over time. The selected age range reflects the pediatric population routinely managed within the study setting and captures the period during which asthma and pneumonia contribute substantially to respiratory morbidity. Second, a multidimensional evaluation of determinants of respiratory diseases through the integration of clinical records with environmental monitoring data and socioeconomic indicators was complete. It allowed the assessment of both environmental exposures and socioeconomic inequalities as well as healthcare usage patterns simultaneously. Third, the presence of both inpatient and outpatient data made certain that the entire gamut of severity in asthma exacerbations, including mild ones and serious pneumonia necessitating hospital stay was covered. Lastly, the analytical rigor was enhanced by the multivariate regression models and time-to-event analysis that were used to determine the independent predictors of hospitalization and recurrent asthma episodes.

### 4.2 Limitations of the study

Even though it has its merits, there are a number of limitations to be taken into consideration when understanding the findings. The retrospective design was based on regularly obtained clinical and administrative data, which could be incomplete or misclassified diagnoses. The estimates of environmental exposure have been based on the regional surveillance data and associated with residential locations, which might not be sufficient to reflect individual-based variation of exposure or indoor microenvironmental factors. Also, some of the possible confounders, including genetic predisposition, vaccination status, nutritional status, and detailed behavioral factors, were not always available in the medical records and, thus, could not be factored in the analysis. The socioeconomic indices were calculated using area rates and not on household measurements, and this could cause some ecological bias. Moreover, since the study was carried in a specific geographic area, there is a possibility that the results cannot be extrapolated to other groups or healthcare systems.

Several household environmental variables were obtained from routinely collected assessment records rather than direct prospective measurements. Although missingness was low and quality-control procedures were applied, some exposure misclassification cannot be excluded. Consequently, the magnitude of observed associations may have been affected by residual measurement error.

The routinely collected electronic medical record system had no data on the older children and adolescents’ smoking status. While the overall prevalence of active smoking is low among children in the age groups studied, and especially the younger children, smoking behavior among 10–14-year-old adolescents may have an impact on respiratory outcomes and hospitalization risk. Adjusting for active smoking may thus have caused residual confounding, and may have slightly affected the estimated associations between environmental exposures and respiratory disease outcomes.

### 4.3 Future recommendations

Prospective longitudinal studies with individual measurements of environmental exposure and more thorough household evaluations are needed to gain a more comprehensive understanding of the causal mechanisms linking environmental determinants, socioeconomic determinants, and the outcome of respiratory diseases in children. The exposure assessment could be more precise by integrating air quality monitoring with wearable exposure sensors or geospatial modeling. Besides that, research investigating preventive measures, including enhanced indoor air ventilation, reduced biomass fuel consumption, and communal air pollution management, would be highly useful for interventions in national health. Additional research into the early-life determinants, vaccination coverage, and genetic susceptibility can also be used to determine high-risk pediatric groups. Regarding health systems, studies evaluating targeted asthma management programs and early intervention strategies would be useful for reducing recurrence rates and healthcare utilization.

Children who live in socioeconomically disadvantaged communities should be targeted by public health interventions, including increasing access to primary health care services, expanding health insurance coverage, implementing school-based respiratory health screening programs, providing health education for parents and children, and reducing household exposure to indoor air pollution. Interventions should be implemented through a partnership among local health authorities, healthcare institutions, and education and environmental regulatory authorities.

Environmental interventions should target specific measures to reduce children’s exposure to traffic-related NO_2_ and PM_2.5_ by optimizing traffic emissions management around residential and school areas, monitoring air quality, promoting cleaner household energy sources, and improving household ventilation.

## 5 Conclusion

Asthma and pneumonia remain to have a significant clinical and healthcare impact on children with specific patterns of healthcare use and recurrence. Environmental pollutants, indoor smoke exposure, and socioeconomic disadvantage were important predictors of hospitalization risk, and seasonal patterns determined the incidence of the disease. These findings underscore the importance of a comprehensive public health approach to decrease children’s exposure to PM_2.5_ and NO_2_, improve household air ventilation, decrease indoor biomass fuel and tobacco smoke exposures, and improve access to healthcare services for socioeconomically disadvantaged families. These should be spearheaded by the local public health authority, health care providers, schools, and environmental agencies via specific strategies like reducing emissions from traffic in residential and school zones, regular monitoring of PM_2.5_ and NO_2_, programs to promote alternative household energy sources, home ventilation improvement campaigns, health education campaigns, and access to health care programs that are both fair and accessible. Strengthening preventive strategies and implementing targeted interventions addressing both environmental exposures and socioeconomic barriers may help reduce the burden of childhood respiratory diseases and improve long-term respiratory health outcomes.

## Data Availability

The raw data supporting the conclusions of this article will be made available by the authors, without undue reservation.
